# Supplementary material for: Circulatory Shock among Hospitalized Patients for Salicylate Intoxication
Source: Diseases. 2021 Jan 12;9(1):7. doi: 10.3390/diseases9010007 (PMC7839009; doi:10.3390/diseases9010007)
Supplement: Supplementary file 1 [file diseases-09-00007-s001.pdf]

Table S1 ICD 9 CM codes

Salicylate poisoning	965.1
Alcohol drinking	291.0, 291.1, 291.2, 291.3, 291.4, 291.5, 291.8, 291.81, 291.82, 291.89, 303.00-303.03, 303.90-303.93, 305.00-305.03
Obesity	278.0, 278.00, 278.01, 649.10– 649.14, 793.91, V85.30–V85.4, V85.54
Anemia	283.00-285.9
Diabetes Mellitus	249.00–249.31, 250.00–250.33, 648.00–648.04, 249.40–249.91, 250.40–250.93, 775.1
Hypertension	401.1, 401.9, 642.00–642.24, 401.0, 402.00– 405.99, 437.2, 642.10–642.24, 642.70–642.94
Dyslipidemia	272.xx
Coronary artery disease	412.xx, 413.xx, 414.xx
Congestive heart failure	428.xx
Atrial flutter/fibrillation	427.31, 427.32
Chronic kidney disease	585.1, 585.2, 585.3, 585.3, 585.4, 585.5, 585.6, 585.9
Liver cirrhosis	456.xx, 567.23, 571.xx, 789.59
Volume depletion disorder	276.5, 276.51, 276.52
Sepsis	003.1, 003.21, 020.2, 022.3, 036.0, 036.1, 036.2, 036.3, 036.42, 038.0, 038.10, 038.11, 038.19, 038.2, 038.3, 038.4, 038.40, 038.41, 038.42, 038.43, 038.44, 038.49, 038.8, 038.9, 098.82, 098.84, 098.89, 112.5, 112.81, 112.83, 115.04, 115.14, 115.94, 117.9, 785.52, 790.7, 995.92
Seizure	345.00-345.91, 780.3-780.39, 89.14
Gastrointestinal bleeding	456.0, 456.20, 530.82, 531.00-531.41, 531.60, 531.61, 532.00-532.41, 532.60-532.61, 533.00-533.41, 533.60, 533.61, 534.00-534.41,534.60, 569.3, 578.0, 578.1, 578.9
Ventricular arrhythmia /Cardiac arrest	427.1, 427.41, 427.5
Gastric lavage	96.07-96.08, 96.33-96.36
Non-invasive ventilation	93.90
Invasive mechanical ventilation	96.70-96.73
Blood transfusion	99.00-99.07
Hemodialysis	39.95, v45.1, v56.0, v56.1
Peritoneal dialysis	54.98, v56.2, v56.32
Acute kidney injury	584, 584.5, 584.6, 584.7, 584.8, 584.9 (exclude 585.5, 585.6)
Respiratory failure	518.81, 518.82, 518.85, 786.09, 799.1, 96.7, 96.70, 96.71, 96.72
Circulatory failure	458.8, 458.9, 785.5, 785.50, 785.51, 785.52, 785.59, 796.3
Liver failure	570, 572.2, 573.3, 573.4
Neurological failure	293, 293.0, 293.1, 293.8, 293.81, 293.82, 293.83, 293.84, 293.89, 293.9, 348.1, 348.3, 348.30, 348.31, 780.01, 780.09, 48.39, 89.14
Hematological failure	286.6, 286.7, 286.9, 287.49, 287.5
